# Novel CYLD pathogenic variant associated with multiple cylindromas of the scalp—A case report

**DOI:** 10.1002/ski2.436

**Published:** 2024-08-10

**Authors:** Inês P. Amaral, Ivânia Soares, Madalena P. Correia, Catarina Correia, Pedro Vasconcelos, Luis S. Almeida, Paulo Filipe

**Affiliations:** ^1^ Dermatology Department Unidade Local de Saúde – Hospital de Santa Maria Lisbon Portugal; ^2^ Faculty of Medicine University of Lisbon Lisbon Portugal

## Abstract

Cylindromas are rare, benign adnexal neoplasms primarily found on the scalp and face, with a higher incidence in women and typically manifesting in the second or third decade of life. These tumours can present as single or multiple nodules, with the latter form often linked to CYLD gene mutations, particularly in the context of CYLD cutaneous syndrome. Here, we report a case of a 61‐year‐old male presenting with multiple cylindromas of the scalp, prompting genetic testing that revealed a novel pathogenic variant in the CYLD gene.

## Graphical abstract

Cylindromas are rare, benign adnexal neoplasms primarily found on the scalp and face, with a higher incidence in women. We report a 61‐year‐old male with multiple scalp cylindromas, leading to genetic testing that identified a novel pathogenic variant in the CYLD gene, highlighting the importance of genetic testing in these cases.

## 1 CASE REPORT

Cylindromas, though rare, are benign adnexal neoplasms predominantly found on the scalp and face, exhibiting a higher incidence among women and often manifesting in the second or third decade of life. These tumours can occur as single or multiple nodules, with the latter form being linked to mutations in the CYLD gene, particularly in the context of the CYLD cutaneous syndrome, an autosomal dominant disorder characterised by the presence of multiple cylindromas, trichoepitheliomas and spiradenomas.^1^ While cylindromas are typically benign, it is crucial to recognise their potential for malignant transformation, which occurs in 5%–10% of the cases.^2^

**FIGURE 1:**
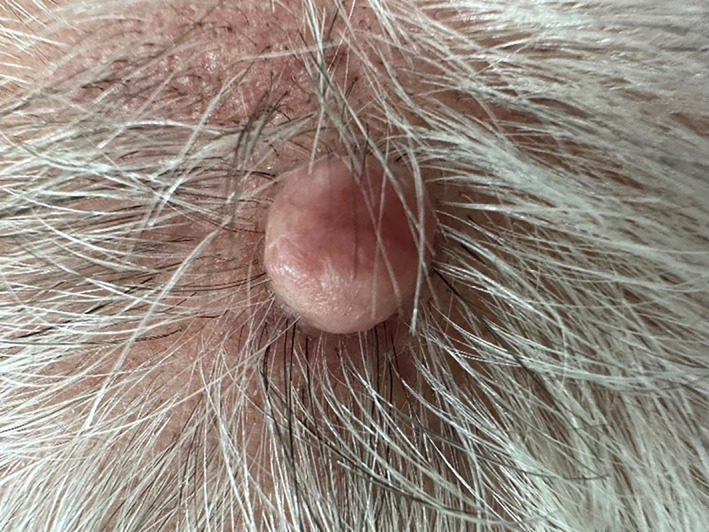
Clinical image of cylindroma, a dome‐shaped, pink to skin‐coloured nodule on the patient's scalp.

**FIGURE 2:**
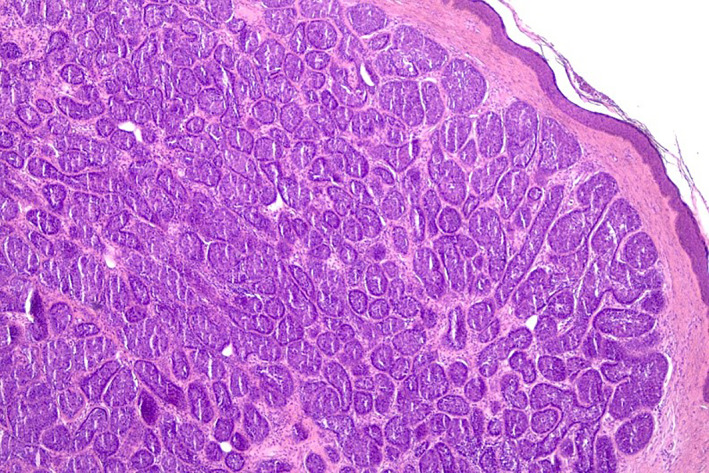
Histopathologic picture of cylindroma, a well‐circumscribed dermal tumour with basaloid cell islands surrounded by a thick, eosinophilic basement membrane disposed like a jigsaw puzzle (C–H&E, ×40; D–H&E, ×100).

A 61‐year‐old male, otherwise healthy, presented with a 20‐year history of multiple cutaneous tumours on his scalp. Notably, a family history revealed similar lesions in his mother and sister, beginning around the age of 20. Upon clinical examination, multiple asymptomatic, dome‐shaped, pink to skin‐coloured nodules were observed across the patient's scalp (Figure 1). Histopathological examination confirmed the diagnosis of cylindroma, demonstrating characteristic features such as well‐circumscribed dermal tumours with basaloid cell islands surrounded by a thick, eosinophilic basement membrane disposed like a jigsaw puzzle (Figure 2).

Genetic testing unveiled a novel heterozygous pathogenic variant in the CYLD gene (c.1124G>A, p.(Trp375Ter)), resulting in the substitution of tryptophan with a premature termination codon. This nonsense variation led to protein truncation and was classified as probably pathogenic. It is worth noting that this mutation has not been previously reported in the literature or databases, indicating its novelty and underscoring the importance of continued genetic research in this field.

The identification of this novel CYLD pathogenic variant expands the spectrum of genetic alterations associated with cylindromas and highlights the importance of genetic testing in patients with multiple adnexal tumours. Understanding the genetic basis of cylindromas can facilitate precise diagnosis, risk evaluation, genetic counselling and tailored management approaches. Moreover, individuals identified with specific genetic mutations may qualify for forthcoming clinical trials investigating non‐surgical pre‐emptive interventions aimed at halting tumour progression in this potentially disfiguring condition.^3^ However, it is worth noting that as of now, no definitive genotype–phenotype correlation has been established.^4^

We present a rare case of multiple cylindromas of the scalp associated with a novel CYLD pathogenic variant. This case underscores the value of genetic testing in patients with suspected familial cylindromatosis and emphasises the need for continued research to unravel the genetic underpinnings of this condition.

## CONFLICT OF INTEREST STATEMENT

The authors declare no conflicts of interest.

## AUTHOR CONTRIBUTIONS

**Inês P. Amaral**: Conceptualization (lead); data curation (equal); project administration (equal); writing—original draft (lead). **Ivânia Soares**: Conceptualization (supporting); validation (supporting); writing—review and editing (supporting). **Madalena P. Correia**: Conceptualisation (supporting); writing—review and editing (supporting). **Catarina Correia**: Conceptualisation (supporting); resources (lead). **Pedro Vasconcelos**: Conceptualization (supporting); resources (lead); writing—review and editing (supporting). **Luis S. Almeida**: Resources (lead). **Paulo Filipe**: Conceptualization (supporting); resources (supporting); supervision (lead); validation (lead); writing—review and editing (lead).

## ETHICS STATEMENT

All procedures performed in this study involving human participants were in accordance with the ethical standards of the institutional and/or national research committee.

## PATIENT CONSENT

Written patient consent for publication was obtained.

## Data Availability

The data that support the findings of this study are available from the corresponding author upon reasonable request.
